# Does climate-smart agriculture improve household income and food security? Evidence from Southern Ethiopia

**DOI:** 10.1007/s10668-023-03307-9

**Published:** 2023-05-12

**Authors:** Abrham Belay, Alisher Mirzabaev, John W. Recha, Christopher Oludhe, Philip M. Osano, Zerihun Berhane, Lydia A. Olaka, Yitagesu T. Tegegne, Teferi Demissie, Chrispinus Mutsami, Dawit Solomon

**Affiliations:** 1 Center for Development Research (ZEF), University of Bonn, Genscherallee 3, 53113 Bonn, Germany; 2 Department of Earth and Climate Sciences, Faculty of Science and Technology, University of Nairobi, P.O. Box 30197-00100, GPO, Nairobi, Kenya; 3 Center for African and Asian Studies, Addis Ababa University, P.O. Box 1176, Addis Ababa, Ethiopia; 4 Stockholm Environment Institute-Africa, World Agroforestry Centre, P.O. Box 30677, Nairobi, Kenya; 5 Climate Prediction and Applications Center Institution, Intergovernmental Authority on Development, Hargeisa, Somaliland; 6 International Livestock Research Institute (ILRI), P.O. Box 30709-00100, Nairobi, Kenya; 7 European Forest Institute (EFI), Platz Der Vereinten Nationen 7, 53113 Bonn, Germany; 8 Department of Geoscience and the Environment, The Technical University of Kenya, Nairobi, Kenya

**Keywords:** Climate change adaptation, Climate-smart agriculture practices, Food security, Farm income, Propensity score matching approach, Southern Ethiopia

## Abstract

Climate change threatens African countries’ economic development and affects agriculture and food security. Ethiopia is especially vulnerable to the negative effects of climate change because its economy is dependent on climate-sensitive livelihoods that have limited potential for adaptation. Emerging evidence indicates that climate-smart agriculture (CSA) can help smallholder farmers adapt to climate change and increase agricultural productivity, thereby enhancing household income and food security. In the study area, different CSA practices have been adopted to mitigate the negative effects of climate change and improve agricultural productivity, income, and food security. Therefore, this study examines the impact of CSA practices on household income and food security in southern Ethiopia. A total of 385 households were selected using multistage sampling. Primary and secondary data were used, and propensity score matching with different types of matching algorithms, such as nearest neighbor, kernel, and radius matching, was employed to quantify the conditional impacts of CSA intervention on farm income and food security. In comparison with non adopters farmers that have adopted CSA practices had a higher food consumption score between 6.27 and 8.15, which was statistically significant at the 1% level. Overall, 34.55% of interviewed households had acceptable food consumption scores, 44.68% had borderline, and 20.77% had poor food consumption scores. Furthermore, households that adopted CSA practices had a 20.30% higher average annual farm income per hectare than non-adopters. The study suggests that effective extension services, accurate climate information, and sound policy support are required to promote and scale up CSA measures in the study area to improve farmers’ adaptive capacity, farm income, and food security.

## Introduction

Climate change (CC) and variability continue to be major global challenges for humanity (Abdallah et al., 2019; Hundera et al., 2019; Pörtner et al., 2022). The negative consequences are especially dangerous for developing countries whose economies rely on climate-sensitive livelihoods with limited adaptation capacity (Asfaw et al., 2021; Zougmoré et al., 2021). For example, the Fifth Assessment Report of the IPCC’s Working Group II (Edenhofer, 2015) stated that the effects of climate change are expected to exacerbate poverty in most low-income countries. This is especially true for rural Africa where people face hunger and food insecurity (Crippa et al., 2021). Climate change has already harmed African agriculture and food security (Njeru et al., 2016). According to scientific evidence, a comprehensive approach is required to successfully develop agricultural systems that foster adaptation and mitigation (Crippa et al., 2021). Most African countries rely on agriculture as their primary economic activity, which is characterized by an overreliance on rainfall and primarily practiced by smallholders with low input use (Mekonnen et al., 2021). Furthermore, a poor land tenure system, low soil fertility, a lack of extension services, extreme weather, land degradation, the COVID-19 pandemic, and rising conflicts exacerbate the situation (Giller et al., 2021; Issahaku & Abdulai, 2020; Zougmoré et al., 2021). Thus, increasing farmers’ awareness of climate change and implementing innovative adaptation measures are critical for mitigating the negative effects of climate change (Khonje et al., 2018). High levels of poverty, food insecurity, and low productivity in Sub-Saharan Africa, including Ethiopia, are largely driven by low levels of agricultural technology adoption and CC-related impacts (Stuch et al., 2021).

Available scientific evidence shows that in response to CC impacts and to ensure agricultural productivity, poverty reduction, and achieve food security, a mix of agricultural practices, such as soil and water conservation measures, agroforestry, and irrigation schemes, have been employed by Ethiopian smallholder farmers (Di Falco & Veronesi, 2018). Additionally, a multi-stakeholder approach involving extension agents, practitioners, policymakers, scientific communities, and other pertinent actors is necessary for the effective development of agriculture toward a climate-smart approach. By adopting climate-smart agriculture (CSA) practices, improving food security and household income, and reducing carbon emissions, the Climate Resilient Green Economy strategy document aims to address critical CC challenges. Furthermore, to mitigate the negative effects of climate change on agricultural production, the Ethiopian government launched a massive campaign to promote cereal crop row-planting through the Agricultural Transformation Agency, in which approximately 2.5 million farmers participated (Fentie & Beyene, 2019). However, farmers may lack sufficient information about the nature of new climate extremes, the resulting problems, and the climate-smart technologies required to deal with them effectively. Consequently, their responses may be inadequate, ineffective, or unsustainable.

In light of this, the CGIAR Research Program on Climate Change, Agriculture, and Food Security (CCAFS), in collaboration with other stakeholders, has been working with farmers in East Africa to test and promote a portfolio of technological and institutional CSA options to deal with climate change in agriculture through the climate-smart villages (CSVs) approach and to scale the right options (Ogada et al., 2020). The CSVs approach has clearly demonstrated its effectiveness in scaling up climate-smart agriculture practices (Aggarwal et al., 2018). According to Aggarwal et al. (2018), between 2012 and 2020, CSV-related outcomes were reported across five CCAFS regions: Southeast Asia (16), South Asia (14), East Africa (12), West Africa (12), and Latin America (10). The adoption rate of improved agricultural technology, performance, and productivity in Ethiopia’s agriculture sector remains inadequate due to a lack of information on the technicality of available agriculture technologies and an insufficient supply of inputs (Berha, 2022; Suri & Udry, 2022; Yu & Nin-Pratt, 2014). A recent study found that CSA practices, such as improved crops, are being used in Africa to increase household income and food security (Ogunyiola et al., 2022; Suri & Udry, 2022). CSA incorporates three dimensions of sustainable development (social, economic, and environment) and focuses on increasing agricultural production and community resilience and, if possible, reducing carbon emissions. For example, Aggarwal et al. (2018) reported that adopting CSA practices increases agricultural productivity, which can improve household income and food and nutrition security. However, empirical research on the impact of CSA practices on smallholder farmers’ income and food security in Ethiopia is insufficient.

Previous studies assessed the effects of CSA practices and technology in Africa in reducing the negative effects of climate change and improving agricultural productivity, income, and food security. Studies examining improved livestock production in Kenya (Ogada et al., 2020) manure composting in Tanzania (Pamuk et al., 2021), improved maize in Zambia (Khonje et al., 2018), improvements in wheat and sorghum production in Ethiopia (Wake & Habteyesus, 2019), silkworm breeding in Rwanda (Habiyaremye, 2017), and soil and water conservation in Somalia (Nyirahabimana et al., 2021) yielded mixed results. For example, Ogada et al. (2020) demonstrated that the adoption of small ruminant livestock breeds reduces farmers’ income. In contrast, Wake and Habteyesus (2019) discovered that adopting CSA practices outpaces traditional farming systems through building resilience and reduce the negative effects of climate change on agricultural production. Furthermore, research on the impact of CSA practices, particularly on income and food security in Ethiopia, has been limited due to the observation that adoption intervention is location-specific (Khoza et al., 2021; Zerssa et al., 2021). The current study aims to bridge this gap in the literature by conducting a focused investigation into the impact of CSA practices on income and food security. In this regard, studies have revealed that CSA intervention in Ethiopia, its impact on local livelihood systems, and the factors influencing CSA adoptions are poorly understood and documented (Fentie & Beyene, 2019; Issahaku & Abdulai, 2020; Mekonnen et al., 2021; Zerssa et al., 2021). Recent studies have examined factors influencing CSA adoption, such as access to climate information, scarcity of agricultural inputs, lack of institutional support, poor extension services, and inappropriate technology (Ogada et al., 2020; Partey et al., 2018; Zerssa et al., 2021). Furthermore, information asymmetry or a knowledge gap may explain the lower CSA adoption rate and its impact on household livelihoods (Asfaw et al., 2021; Ullah et al., 2020). In theory, CSA steadily increases agricultural productivity, strengthens farmers’ resilience to climate change, and reduces greenhouse gas emissions wherever possible (Njeru et al., 2016). Evidence from various East African countries suggests that smallholder farmers’ adoption of CSA practices increases agriculture’s ability to adapt to climate change and improves the well-being of farm households (Bazzana et al., 2022). Several CSA practices have been implemented in the study area to mitigate the negative effects of climate change and increase agricultural productivity. Thus, this study employs quantitative evidence from a cross-sectional survey dataset due to inconsistent findings in the empirical literature and a lack of CSA information on the study area.

The study adds to the existing literature by offering valuable baseline data for further investigation and policy intervention into CSA practices in the Southern region of Ethiopia**.** The remaining sections of the paper present our methodologies, results and discussions, and conclusions and policy implications.

## Conceptual/theoretical framework of the study

Recent studies show that potential risks and uncertainty influence the adoption of new agricultural practices and technologies (Mugabe, 2020; Bazzana et al., 2022). Agricultural technology is a broad concept encompassing equipment, genetic material, farming techniques, and agricultural inputs developed to improve agricultural efficiency. Agricultural technology adoption theory is a multidisciplinary field that attempts to explain why some farmers accept new technologies while others do not by combining elements of decision theory and the diffusion of the theory of innovation (Ruzzante et al., 2021). According to expected utility theory assumptions, farmers that make a utility maximization decision to adopt new agricultural technology, such as CSA practices, based on the risks and potential uncertainties are also subject to farm input constraints (Jaeger, 2007; Mercer, 2004). The decision to maximize utility or profit is a function of farmers’ preferences or selection from available alternatives, which include CSA practices and technologies (Marra et al., 2003; Wens et al., 2021). Adoption theory demonstrates that farmers’ resource allocation decisions for different agricultural practices are subject to maximizing the expected utility of food and income by selecting specific CSA practices under risks and uncertainties (Maina et al., 2020). In this study, for example, smallholder farmers expect benefits or utilities from adopting CSA practices that maximize their income and food security (Sardar et al., 2021). Smallholder farmers adopt new agricultural practices and technology if the expected utility or benefits from adoption (Ua) are substantially greater than those from non-adoption (Un) (Kassie et al., 2015; Ngoma et al., 2021). Following Wooldridge (2010) and Greene (2000), we derived the utility function from the adoption of CSA with dichotomous choices, which are determined by the given observable and unobservable characteristics of *Z*_i_ and error term *ε*_i,_ such that:

1 $$I^{*} i = \beta Z_{i} + \varepsilon_{i} , \, I_{i} = 1\;{\text{if}}\;I^{*} > 0\;{\text{and}}\;0\;{\text{otherwise}}$$

where *I*_*i*_ represents a binary choice variable for CSA adoption, which equals 1 if the farmers *I* adopt the CSA practices, and 0 otherwise. *β* represents the coefficient of the vector parameters to be estimated, *Z*_*i*_ represents socioeconomic characteristic of the farmers, and *ε*_*i*_ is the error term. Therefore, farmers adopt CSA practices if *I*_*i*_ = *Ua* − *Uu* > 0. Hence, the probability of households’ adoption of CSA practices and technology would be quantified as follows:

2 $$\Pr \left( {I_{i} = \, 1} \right)\;{\text{and}}\;\Pr \left( {I^{*} I > 0} \right) = 1 - D\left( {\beta Z_{i} } \right)$$

where (*I*_*i*_ = 1) represents the probability of CSA adoption, and *D* is the cumulative distribution function for the error term (*ε*_*i*_), which differentiates the types of model used for estimation (Greene, 2003).

Adoption of CSA is critical for improving farmer welfare in the face of CC threats (Boz & Shahbaz, 2021; Sardar et al., 2021). Adopting CSA practices and technology can assist smallholder farmers in achieving food security, increased income, and poverty reduction. According to recent research, increasing agricultural yield can improve farmers’ well-being by increasing household income and food security (Hussain et al., 2022; Musafiri et al., 2022; Ogada et al., 2020; Ogunyiola et al., 2022; Warinda et al., 2020).

**Fig. 1:**
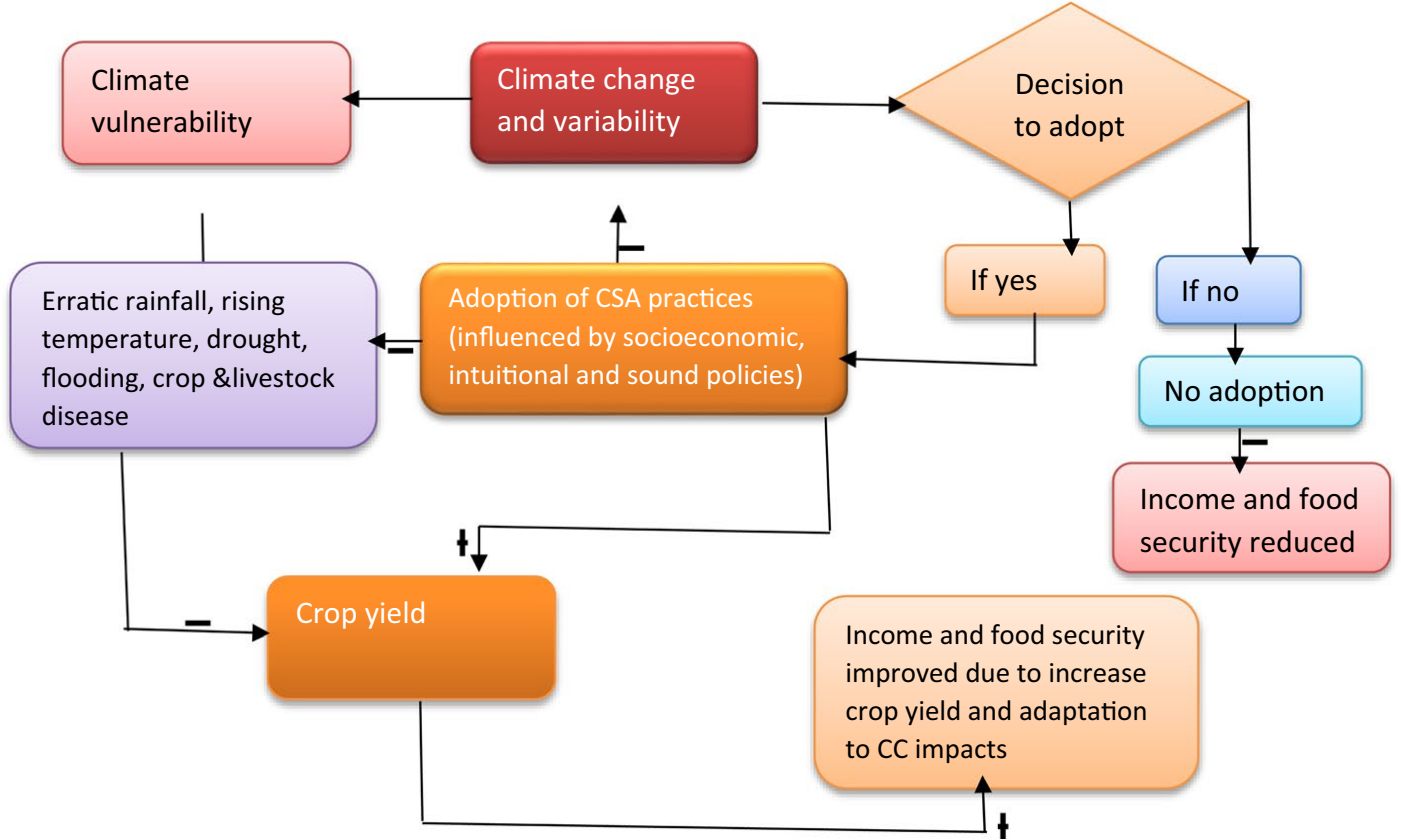
Conceptual formwork of the study. *Source*: Adopted from Sarda et al. (2021)

Given the aforementioned theories and assumptions, the conceptual framework of this study is constructed, which consists of four main components: (1) CC/variability and its effects; (2) adoption of CSA; (3) crop yield increment; and (4) household income and food security. The conceptual framework follows top-down and bottom-up approaches (Fig. 1 Straight lines with positive and negative signs show the positive and negative effects that one part of the framework has on the other parts of the framework, respectively). Due to their vulnerability to climate-related risks and limited capacity for adaptation, smallholder farmers’ livelihoods are negatively impacted by climate change and variability (Ogada et al., 2020).

Smallholder famers can reduce climate-related impacts by adopting CSA practices and technology (Warinda et al., 2020). Farmers’ intentions to use CSA practices depend on how well they understand and know about climate change and how vulnerable they are (Sardar et al., 2021). Hence, the effective and timely adoption of CSA practices in farming depends on knowledge and perception regarding CC and its impact (Boz & Shahbaz, 2021). In addition, the intention of farmers to adopt available CSA practices is influenced by experience, knowledge, skills socioeconomic and intuitive support, infrastructure, and sound policies (Ruben et al., 2019). In this study, we consider a rational farm household that adopts CSA measures to offset climate-related agricultural losses. A farmer’s adoption of CSA practices not only decreases climatic risk but also enhances crop productivity and farm income by increasing crop production. The benefits of CSA adoption have a direct positive effect on farmers’ income growth and mitigate the adverse effects of CC and variability (Hussain et al., 2022). In addition, Ogunyiola et al. (2022) showed that farmers’ adoption of CSA practices tends to reduce climatic risk, increase crop productivity, improve household income, and meet farmers’ food consumption needs.

Conversely, if farmers choose not to adopt CSA or adopt conventional farming, it may increase risks by making them more vulnerable to climate change and may affect the farm income by lowering crop yields per hectare (ha) (Sardar et al., 2021). Adapting CSA practices can help smallholder farmers increase their crop income and food security while mitigating losses and damages caused by climate change (Musafiri et al., 2022; Sedebo et al., 2022). As a result, the study’s conceptual framework is based on CC, its implications for farmers’ agricultural practices, and the direct impact of CSA adoption on household income and food security.

## Methods

### Description of the study area

**Fig. 2:**
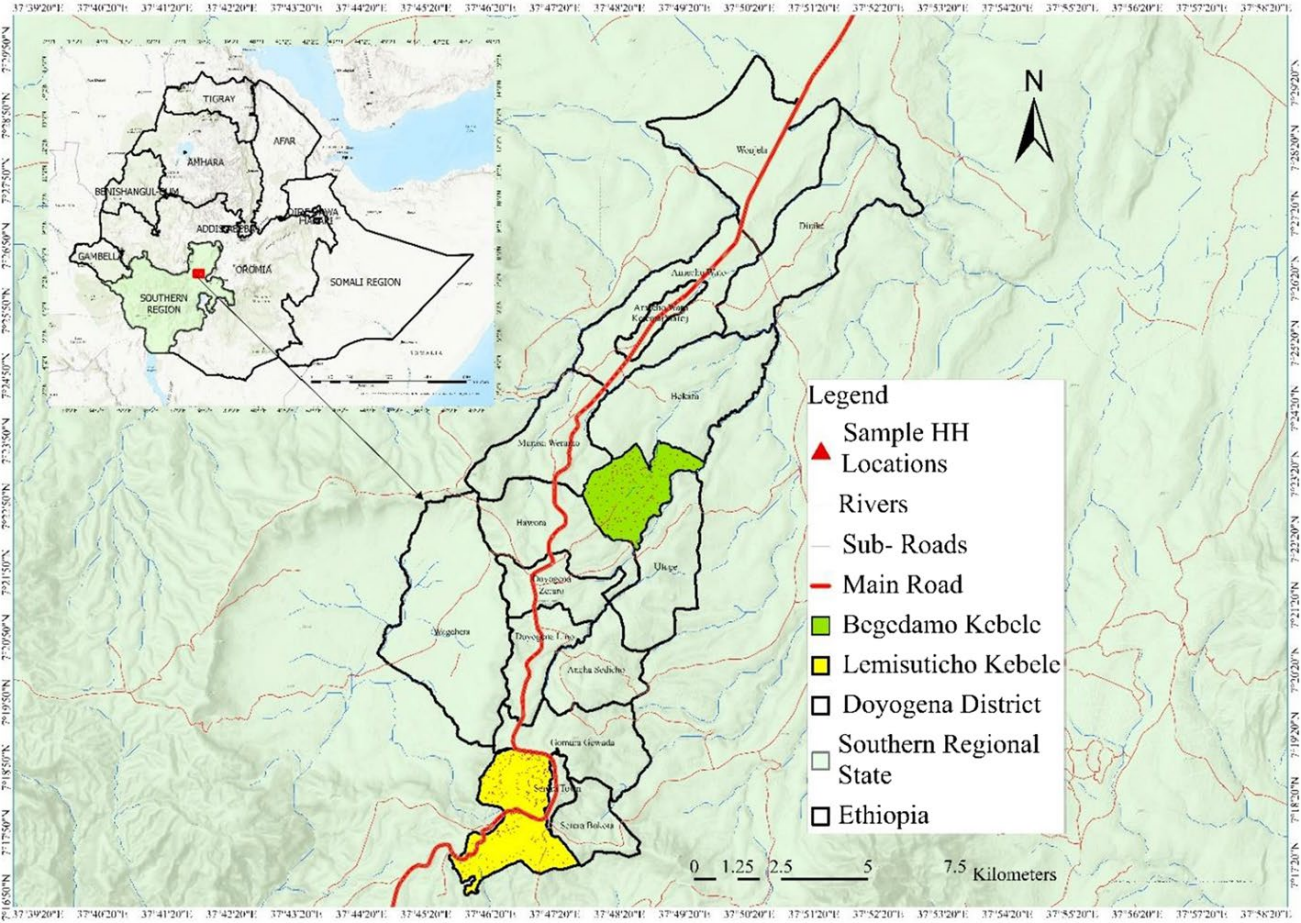
Study area map. *Source*: Author’s own work

This study was conducted in the Doyogena district of the Kembata Tembaro Zone located in the Southern Nations Nationalities and Peoples Region of Ethiopia (Fig. 2). The topography is steep, so the area is mostly classified as highland. The steep nature of the landscapes, combined with extreme flooding, causes high soil erosion and soil fertility loss, thus reducing agricultural production and productivity (Belay et al., 2021). The area has a total population of more than 116,000 people, and crop and livestock production is the farmers’ main income source. Crop production alone accounts for roughly 60% of household income, with livestock production accounting for the remainder (Mathewos et al., 2021). The study area was described in detail by Belay et al. (2022).

### Sampling design and data collection techniques

In choosing sample households, the study used multistage sampling techniques. In the first stage, the Southern region of Ethiopia, particularly Doyogena district, was selected due to its transition from conventional to CSA practices. Two sites, Lemisuticho and Begedamo *kebele*, from the district were selected in the second stage, and these two sites are assumed to have similar topography, planting and harvesting seasons, and crop and livestock livelihood systems. In the third stage, eight villages were randomly selected with the help of extension workers and peasant association leaders. Four villages from the Lemisuticho *kebele*, namely, Tula, Suticho, and Cherema Kanko, and four villages from the Begedamo *kebele*, namely, Gateme, Bakucho, Tach-begedamo, and Lay-begedamo, were selected. These villages represent areas where CSA and conventional agriculture practices are being practically implemented. The sample households were selected from each village proportional to the sample size using the rand between function in Microsoft Excel (Mesfin et al., 2020).

Meanwhile, using Cochran’s formula (Heinisch, 1965), this study selected 385 households from 10,267 sampled populations, with 80.5% male and 19.5% female respondents.

3 $$n = \frac{{Z^{2} pq}}{{e^{2} }} = \frac{{(1.96^{2} )\left( {0.5} \right)\left( {0.5} \right)}}{{(0.05)^{2} }} = 385\;{\text{house}}\;{\text{holds}}$$

where *n* indicates the sample size for the study *p* = estimated variance in the population, *q* = 1 − *p*, *Z* = Z-score at the desired confidence level, *e* is the acceptance error (5%) at 95% level of confidence, and *Z* = 1.96.

The household survey was administered by research assistants and enumerators who were fluent in the local language and had participated in the prior interview exercise. Focus group discussions, key informant interviews, semi-structured questionnaire surveys, and direct observation were used to collect quantitative and qualitative data (Creswell & Creswell, 2017). Before actual data collection, the questionnaire and checklist were pretested to ensure that they were correct and valid. Prior to interviewing the sample households, 32 non-sample households were identified and interviewed to pretest the questionnaire. This allowed restructuring the questions before extensive data collection. The questionnaires and checklists were then amended and enriched for the actual interview based on the constraints identified in the pretest. This was critical to capture farmers’ understanding of the questions and obtain accurate information.

The information contained in the data collected from primary and secondary sources included socioeconomic characteristics of the farmers, CC conditions, farmers’ income sources, and food security. Farmers’ income data were gathered using the expenditure method that is less sensitive to measurement than direct annual estimation. Farmers were asked, “What have been your monthly expenses for food, agriculture inputs, medication, closing, and so on?” In this case, the basic needs approach was used to estimate farmer expenditure, which was aggregated from food (measured over a week and then multiplied by 4.1 to convert to monthly food expenditure) and non-food expenditure for essential products estimated monthly. Furthermore, the estimated monetary value of food and non-food expenditures is calculated using consumer price index data obtained from the Central Statistical Services (Battistin, 2003; Deaton, 1997; Kanu & Okezie, 2021). Secondary data were also gathered from a variety of sources, including published articles and policy-related working documents.

### Analytical framework and model specification

Agricultural investment using new farming technologies looks attractive to smallholder farmers if they perceived that the benefits outweigh the costs. The farmers’ decision to adopt CSA practices may be viewed as constrained optimization if the practices are either available, affordable, or advantageous. Smallholder farmers are assumed to be rational in adopting CSA practices that meet their needs and enable them to overcome the challenges posed by climate variability in their farming endeavors. In this study, household income and food security were used to evaluate the impact of CSA practices and technologies. The household’s income was derived from on-farm (crop-livestock) and off-farm sources, including remittances, self-employment income, agricultural income, and non-agricultural wages (Bojnec & Knific, 2021).

Household food security is a condition in which all household members have access to sufficient, nutritious, and safe food to meet their dietary needs at all times (Tendall et al., 2015). Food Consumption Score (FCS) was found to be an effective indicator for measuring the number of food groups consumed in a household during a given reference period, as frequently employed by the World Food Program (Mujeyi et al., 2021). The FCS is used extensively to indicate dietary diversity when assessing food security. It is computed based on the frequency of weighted dietary diversity of households consuming eight food groups (i.e., cereals, pulses and nuts, vegetables, fruits, meat and fish, dairy products, sugar_honey and oil fat_butter and other condiments) with a 7-day recall from the date of survey data collection. The consumption frequency is then summed to give the food group score, and each group score is multiplied by the weights of each nutrient density of the given food group to create the FCS (Carletto et al., 2013), as shown in the following:

4 $${\text{FCS}} = \sum y_{i} f_{i}$$

where FCS is food consumption score, *y*_*i*_ represents different food groups, *i* is the weight of the nutritional value of each food group, and *fi* is the consumption frequency of food groups that the households consumed within the last 7 days.

Adopting CSA options help farmers increase food supply availability and generate more income. Smallholder farmers decide to adopt a new farming strategy in this process and estimating the impact of such adoption intervention is critical (DiPrete & Gangl, 2004). To estimate the impact of CSA adoption on household income and food security outcome measures, this study used the propensity score matching (PSM) method (Brüssow et al., 2017; Khonje et al., 2015). Previous studies used PSM to compare the outcomes of individual farm households with their counterfactual by controlling for observable farm characteristics and self-selection bias to solve matching problems and estimate the average treatment effect (ATE) of technology adoption (Brüssow et al., 2017; Rosenbaum & Rubin, 1983). PSM estimation helps investigate the impact of CSA adoption under the assumptions of conditional independence (unobserved factors cannot influence CSA adoption) and common support (propensity score overlaps among adopters and non-adopters) (Maina et al., 2020).

In pre-treatment observable characteristics, the PSM approach is used to find a similar group of farmers who have adopted the available CSA options and compare them to other similar farmers who have not adopted any CSA options. After controlling for the pre-treatment observable characteristics associated with CSA adoption, we confirmed that adopters and non-adopters have similar outcomes as non-adopters would have had they adopted CSA. This study’s outcome variable includes income and food security. The outcomes (food security or household income growth) of individual farmers who participated in CSA practices (*y*1) are compared with that of similar farmers who did not participate in CSA adoptions (*y0*), which serves as the basis for the ATE.

The average gain from the result of program participants (treatment group) versus non-participants (control group) can be introduced as follows:

5 $${\text{ATE}} = E\left( {Y_{i} \left( 1 \right)|T_{i} = 1} \right) - E\left( {Y_{i} \left( 0 \right)T_{i} = 1} \right)$$

where *Yi* is the outcome for individual *i*, *T*_*i*_ is the treatment dummy variable, *Y*_*i*_ (1) represents an outcome of individual under treatment, and *Y*_*i*_ (0) is an outcome of individual who is nonparticipant.

PSM constructs the statistical comparison group based on the likelihoods of participating in the treatment (in our case, adoption of CSA practices), conditional on variables (covariates) that are thought to affect treatment participation and can be expressed as follows:

6 $$P\left( X \right) = \Pr \left( {T = 1|X} \right)$$

Food security and gross household income are the study’s outcome variables, which measures the effects of CSA adoptions. The outcome of farmers who adopted CSA practices in response to the perceived effects of CC is contrasted with those of farmers who did not adopt any CSA measures; these two groups are assumed to be not different systematically besides CSA adoption. In this regard, the ATE on treated (ATT) and untreated (ATU), respectively, measures the changes in the outcomes of the treatment and control groups (food security or household income) after matching.

Therefore, the ATT is the difference between with and without treatment, which is given by:

7 $${\text{ATT}} = E\left( {Y1|T = 1} \right) = + E\left[ {\left( {Y1|T = 1} \right)} \right] - E\left[ {Y\left( 0 \right)T = 1} \right]$$

Two presumptions, that is, common support condition and conditional independence, underlie the validity and satisfaction of the PSM estimation outputs (Rosenbaum & Rubin, 1983). The conditional independence or unconfoundedness assumption states that the potential outcome is independent of treatment status and used to establish an unbiased counterfactual for the treatment group after controlling the set of Xi observable covariates (Wake & Habteyesus, 2019). The equation can be expressed as follows: (*Y*_*i*_^*T*^, *Y*_*i*_^*C*^)┴*T*_*i*_|*X*_i_*. Then, we obtain:

$$Y_{i} \left( 1 \right) = Y_{i}^{T} \;{\text{and}}\;Yi\left( 0 \right) = Y_{i}^{C}$$

where *Y*_*i*_^*T*^ is the outcome of an individual on treatment, *Y*_*i*_^*C*^ is the outcome of an individual on control, *X*_i_* is the covariate, and ┴ is the independence. Meanwhile, the assumption of common support certifies that as overlapping between treated and untreated groups is sufficient, acceptable matches can be obtained. The equation is given as 0 < 

$$P\left( {T = 1|X} \right) < 1.l$$

It guarantees that the comparison observation is close to the treatment observation in the propensity score distribution. According to Rosenbaum and Rubin (1983), the treatment assignment is intended to be strongly ignorable if the presumptions are true. In principle, PSM estimation consists of two stages. The first stage is based on the whole sample and a binary outcome model, which is a probability conditional on the characteristics of the households, with a binary treatment variable. This stage also reduces section bias using a matching algorithm as a robustness check (Caliendo & Kopeinig, 2008). Based on the observable characteristics *X*, PSM calculates farmers’ likelihood to participate in CSA practices. It then generates the propensity score *P*(*X*) by creating comparison matching groups with comparable propensity scores, and unmatched units are eliminated from the model (Caliendo & Kopeinig, 2008).

Different matching algorisms commonly used in PSM estimation include nearest neighbor matching (NNM), Kernel-based matching method, radius matching, and caliper matching. In the second stage of PSM, the impacts of CSA adoption on the average outcome variables (food security and income) were determined, and the ATT was estimated. The NNM method is used to pair participant households with nonparticipant households based on their propensity score distance. However, the NNM faces bad matches if the propensity score distance between two neighbors is large. This can be avoided by imposing a tolerance level on the maximum propensity score, and caliper matching with 0.1 restrictions was specified for common support conditions (Smith & Todd, 2005). The kernel matching method constructs the counterfactual outcome and produces the ATE on the treated using the kernel weighted average of the farmers in the adopter group.

The key capability of PSM estimation is controlling selection bias, which is dependent on two conditions: the balancing performance of the given covariates and the absence of systematic household heterogeneity due to the household’s unobserved characteristics (Caliendo & Kopeinig, 2008). The balancing test in PSM estimation assumes that it will balance the variable distribution, reduce bias, and eliminate potential differences in the given covariates (Rosenbaum & Rubin, 1983). Moreover, the PSM addresses the systematic difference due to observable characteristics among households in two ways: first, by estimating the probability of propensity score for each observed characteristic using the logit or probit model, and second, by matching each adopter with non-adopters who have the same propensity score value to estimate the ATT.

Aside from PSM, different approaches, including switching regression, conditional Ricardian model, fixed effect generalized least square, the difference in different approaches, two-stage least square methods, and Heckman logit model, have been used in the recent literature to estimate the unbiased ATT in terms of income and food security. The Heckman selection model was employed to resolve the possible correlation between observed covariates and selection bias (Asrat & Simane, 2017). Furthermore, the Rosenbaum bound test could address unobservable hidden bias (Heckman & Navarro-Lozano, 2004; Rosenbaum, 2002). The testing procedure entails changing the bound on the significance level at the ATT under the given assumption of self-selection into CSA adoption, which allows identification of the critical level of ATT estimation that would become insignificant (Caliendo & Kopeinig, 2008). Following Sardar et al. (2021), this study estimates the ATEs by comparing the expected crop income and FCS of CSA adopters and non-adopters (i.e., counterfactual outcomes). The variable description and measurement used in the model are available in Appendix 3.

## Result and discussion

### Descriptive statistics

Table 1 provides a summary of the variables included in the empirical study, which were chosen based on the relevant literature. The descriptive statistics indicate that socioeconomic variables have significant mean variation between CSA adopters and non-adopters. Variables, such as landholding size, education, soil fertility, slope of farm plot, frequency of extension, training received, social membership, total livestock unit climate perception, and farmers’ willingness to take risks with new agri-technologies, exhibit significant difference between adopters and non-adopters of CSA practices. The average level of education of CSA non-adopters and adopters is approximately 2 and 4 years, respectively. Farmers with a higher level of education are more likely to adopt new agricultural technologies (Fentie & Beyene, 2019). In comparison with non-adopters, CSA adopters are perceived to be more aware of CC and willing to adopt new farming technologies on their farms. Moreover, the average demand for family members in the labor force is 4.33 for adopters and 3.25 for non-adopters. The difference between CSA adopters and non-adopters regarding labor demand is statistically significant. According to Asfaw et al. (2012), agricultural practices are naturally labor intensive, and improving agricultural technologies require skilled labor.

**Table 1:** Summary and descriptive statistics of the variables included in the model

Variables	Non-adopters mean (SD)	Adopters mean (SD)	*p*-value
HH crop income log (in ETB)	9933.91 (828.13)	15,209.41 (986.83)	0.002***
HH_FCS (food consumption score)	36.40 (0.434)	43.70 (0.416)	0.001***
Age (years)	52.06 (1.18)	49.41 (0.83)	0.200
Education (years)	2.84 (0.22)	4.55 (0.36)	0.050**
Family size	7.77 (0.19)	7.21 (0.15)	0.130
Labor	3.25 (0.24)	4.33 (0.16)	0.050**
Gender (1 = Male)	0.79 (0.03)	0.81 (0.02)	0.360
landholding size (hectare)	0.54 (0.03)	0.65 (0.03)	0.025**
Farming experience (years)	26.72 (1.24)	25.02 (0.80)	0.320
Distance to output market (km)	0.82 (0.03)	0.89 (0.02)	0.210
Access to climate information (1/0)	0.07 (0.03)	0.86 (0.03)	0.023**
Soil fertility (1/0)	0.63 (0.03)	0.78 (0.02)	0.012**
Slop of_farm plot (1/0)	0.56 (0.04)	0.57 (0.032)	0.130
Number of extension contacts (frequency)	2.02 (0.06)	5.78 (0.06)	0.053*
Training received (1/0)	0.58 (0.04)	0.82 (0.02)	0.005**
access credit (1/0)	0.27 (0.03)	0.28 (0.02)	0.232
Social membership (1/0)	0.66 (0.015)	0.94 (0.014)	0.025**
TLU	2.46 (0.13)	4.46 (0.13)	0.023**
CC perception (1/0)	0.67 (0.021)	0.87 (0.022)	0.001***
CSA adoption _risk (1/0_	0.42 (0.04)	0.72 (0.02)	0.000***
rainfall_var (coefficient of variation)	1.73 (0.07)	2.02 (0.09)	0.001***

The numbers in parenthesis are standard errors. *, **, and *** represent the level of significance at 10%, 5%, and 1%, respectively. At the time of the survey year 2020, 1 USD = 37.35 Ethiopian Birr (ETB)

The perceived soil fertility status indicated that CSA adopters are more likely to have more fertile cultivated land than non-adopters. Anang et al. (2021) and Mwongera et al. (2017) indicated adoption of improved agricultural technologies, such as soil and water conservation with biological measures, agroforestry systems, and residue incorporation (wheat/barely), increases the soil water holding capacity, soil fertility, and carbon sequestration potentials.

In this analysis, the outcome variables are household income and food security status, as measured by the FCS. The gross household income and FCS variables reveal a statistically significant (1%) difference between CSA adopters and non-adopters. Regarding the food consumption score, farmers who have adopted CSA practices tend to have a higher FCS than their counterparts, with mean FCS values of 36.40 and 43.70 for adopters and non-adopters, respectively. Similarly, farmers who have adopted CSA practices have a higher average annual gross income than their counterparts, with a mean annual gross income of 9,933.91 ETB (1 USD = 37.35 ETB) for non-adopters and 15,209.41 ETB for adopters. The *t*-test result indicated a significant difference between adopters and non-adopter for both FCS and annual crop income.

During the focus group discussion (FGD) and key informant interview (KII), farmers were asked a broad question on their feelings about climate change and variability. The replies were used to assess farmers’ perceptions. Participants were asked to provide evidence of the climate change and variations they had noticed or experienced over the last three decades in the follow-up questions. Most farmers who participated in FGD agreed that climate change is already occurring and mentioned how it affects their farming. The findings are consistent with those of a study conducted in Ethiopia’s Central Rift Valley, which indicated that 90.3% of respondents are well-versed in the meaning of climate change (Hundera et al., 2019). Farmers’ responses to climate change and its impacts were sometimes inconsistent, and they were repeatedly asked to substantiate their response about the term “climate change.” The KII from Lemisuticho kebele, who has lived in the area for about 35 years, claimed that 20 years ago, the amount of rainfall was relatively normal and sufficient for planting. However, in the last 10 years, the duration and number of rainy seasons like “*belg*” (small rainy season) and “*meher*” (harvesting season) have got shorter and less predictable. In terms of temperature change, a key informant stated that temperatures have risen over the last two decades. Furthermore, as temperatures have risen, several water springs have dried up and the amount of water in local rivers has become insufficient as compared with the previous two decades. A farmer participating in the KII noted that over the last 5–10 years, the rainfall pattern has been unpredictable and short, with either early or late start rainy seasons. Meanwhile, more dry spells and longer droughts have been observed in the study area (Belay et al., 2021). Hence, farmers’ awareness and perceptions regarding climate change were consistent with the metrological observations of rainfall and temperature trends in the study area (Belay et al., 2021).

Application of CSA practices at the farm level is context-specific and depends on resources availability, institutional factors, and the severity of CC conditions (Keshavarz & Moqadas, 2021). The common CSA practices strategies adopted by the farmers in the study include soil and water conservation with biological measures, crop rotation (cereal/potato), improved crop varieties (high yielding beans, potato wheat), agroforestry systems (wood perennials crops), improved breeds (small ruminants), and residue incorporation (wheat/barely).

Adopting CSA practices boosts agricultural productivity, raises household income, strengthens farmer resilience, and slows CC (Lipper et al., 2014). Tadesse et al. (2021) conducted research in the same study area on the effect of CSA adoption on soil fertility status, crop yield, and soil carbon between CSA adopters and non-adopters using different soil fertility indicators such as total nitrogen (TN), soil organic carbon (SOC), and plant-available phosphorus. The findings show that the adopters’ farm plot’s soil fertility was significantly higher (*p* < 0.05) than that of the non-adopters in the current study area (Tadesse et al., 2021). Tadesse et al. (2021) added that SOC, TN, and phosphorus content in the soil were 2.8–3.1, 2.2–2.6, and 1.7–2.7 times higher under CSA practices than under conventional farming, respectively. Wheat yield production under CSA intervention increased by 30–45% compared with conventional farming and significant at *p* < 0.05, given the soil fertility indicators. Tadesse et al. (2021) added that CSA intervention was carried out across various landscapes (forest, crop, and agroforestry) in the current study area. The SOC stock was measured at a depth of 1 m at both adopters and non-adopters, and the results show that SOC stock increased under forestland, grassland, cropland, and agroforestry by 3.2%, 4.6%, 5%, and 6.9%, respectively, compared to conventional farming. Agroforestry landscapes had the highest SOC stock (312 Mg C ha^−1^), followed by cropland landscapes (229 Mg c ha^−1^).

**Fig. 3:**
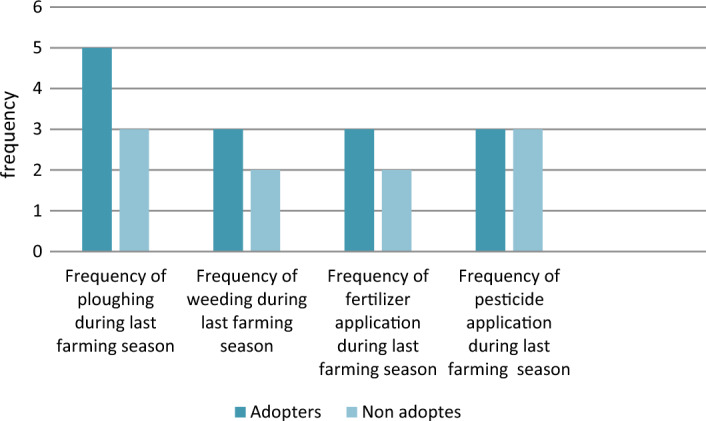
Selected farm management practices in farmers’ farm plot

Previous research indicates that effective CSA adoption at the farm level necessitates productive labor and financial resources that farmers can access, and the ability to combine the resources and implementation (Cinner et al., 2018; Kangogo et al., 2021). Following the adoption of CSA practices, farmers in both the adopter and non-adopter groups were asked about their selective farm management practices and frequency of application (Fig. 3). The results of selected farm management practices (Fig. 3) highlight that CSA practices necessitate a greater frequency of farm management practices and commitment than conventional farming systems. This finding is consistent with Atitianti et al. (2018) and Kangogo et al. (2021), who found that effective CSA adoption at the farm level requires productive labor and resource mobilization.

**Fig. 4:**
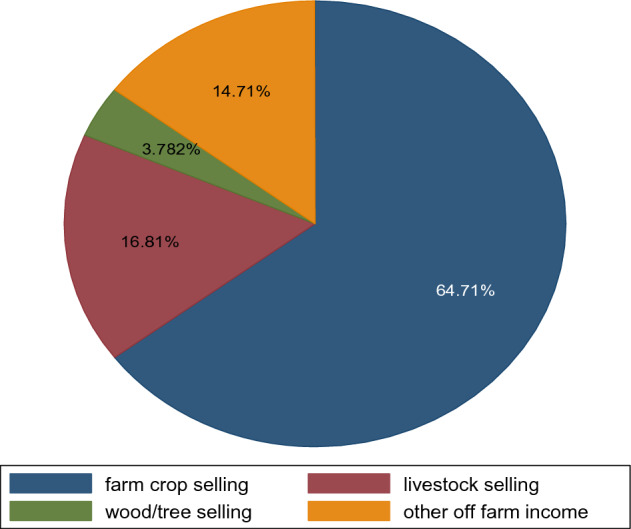
Farmers’ sources of income in the study region

Agriculture is the main source of income in rural communities, and raising agriculture production through CSA practices increases household income and food security (Lipper et al., 2014). Farmers in the study area used multiple agricultural techniques and an approach to income diversification. Innovative agricultural practices directly and indirectly enhance household livelihood outcomes. Examples of the direct effects are high crop and livestock production and a reduction in the cost of production, which improves income and food security. Meanwhile, an increase in food supply that enhances food availability in markets is one indirect effect of innovative agricultural practices. It may be necessary to increase labor demand to increase agricultural labor wages and thus, increase agricultural productivity. The main sources of income for farmers in the study area include farm crops, livestock, tree, and other off-farm means (Fig. 4). Crop selling was the primary source of income and means of subsistence for about 64.71% of farmers. Moreover, farmers in the study area have been producing livestock production like improved breeds (e.g., small ruminants). After crop sales, farmers reported that livestock sales are their second-largest source of income. The outcome is consistent with the findings of Brüssow et al. (2017), who studied the impact of Tanzanian farmers’ adoption of the CSA strategy on food security, and Wordofa et al. (2021), who explored the contribution of agriculture to dietary diversity in Ethiopian households.

**Fig. 5:**
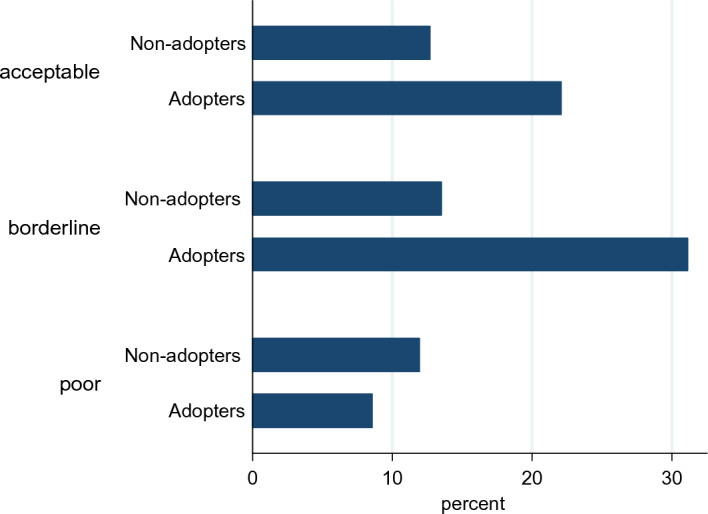
Categories food consumption score

The effects of improved agricultural practices on dietary diversity were analyzed based on crop production and FCS indicators. Farmers in the region under study produce and consume various foods. Figure 5 depicts the percentage of households that fall into various food consumption categories. Household’s FCS measures its dietary diversity by multiplying the frequency of each food group by an assigned weight, resulting in a weighted group value (Maxwell et al., 2014). Sampled households were categorized into poor, borderline, and adequate food consumption categories based on their calculated FCS between CSA adopters and non-adopters. Of the total household, 22.34% of adopters and 12.21% of non-adopters were found in acceptable food consumption categories. In addition, 31.17% of adopters’ households and 13.51% of non-adopters’ households had borderline scores for food consumption. Meanwhile, 8.30% of households in the adopters group and 12.47% of households in the non-adopters group had poor consumption scores. Overall, the average FCS among CSA adopters and non-adapters was statistically significant at the 1% probability level (*p* < 000). The result is consistent with the findings of Aweke et al. (2020) and Teka and Lee (2020), who reported that improved agricultural practices contribute to household welfare in Ethiopia.

### Estimation of propensity score and matching

The STATA statistical package version 14.2 was used for the empirical analysis, and the propensity matching process was carried out by specifying the propensity scores for treatment variables. A binary logit model was used to forecast the likelihood of CSA adoption. The overlapping condition and balancing proclivity were established and met. Based on the minima and maxima criteria, the predicted propensity scores for adopters ranges from 0.108 to 0.987 with a mean of 0.729. Meanwhile, the predicted propensity score for non-adopters ranges from 0.010 to 0.896 with a mean of 0.439. Thus, the common support condition was satisfied in the region [0.108, 0.896], and 68 observations were located outside the common support region. In other words, households with a propensity score less than 0.108 and greater than 0.896 were excluded from consideration for the matching condition.

The NNB, RM, and KM matching algorithms were used to estimate the effect of CSA adoption on household income and food security. Table 2 shows the estimated results of the logit specification of the propensity scores. As shown in Table 2, education, family size, family labor, landholding size, livestock ownership, and perceived soil fertility status are likely to influence the adoption of CSA practices. Access to climate information, annual contact with extension services, climate perceptions, and farm training received are likely to facilitate the adoption of CSA practices. The findings are consistent with previous research on farm-level adaptation to CC in the Ethiopian highlands (Gebrehiwot & Van Der Veen, 2013), the adoption of CC adaptation strategies by maize-dependent smallholders in Ethiopia (Bedeke et al., 2019), CC and farmer adaptation in Sub-Saharan Africa (Kalimba & Culas, 2020), and adoption determinants of multiple CSA technologies in Zimbabwe (Mujeyi et al., 2021).

**Table 2:** Estimated results of the logit specification of the propensity scores

CSA_Adoptions	Coef	Std. Err.	*z*
Age	− 0.026	0.015	− 1.700
Education	0.126	0.037	3.390***
Family size	− 0.148	0.053	− 2.800**
Family labor	0.142	0.052	2.730**
Gender	0.515	0.323	1.590
Landholding size	0.920	0.325	2.830**
Farming experience	0.001	0.015	0.080
Distance market	− 0.076	0.025	− 3.020***
Climate information	0.519	0.132	3.931***
Soil fertility	0.906	0.296	3.060***
Slop_farmplot	− 0.263	0.266	− 0.990
Contact extension	0.029	0.011	2.636**
Training received	1.587	0.290	5.480***
Access to credit	− 0.104	0.281	− 0.370
Social member	− 0.606	0.663	− 0.910
TLU	0.47	0.070	2.428**
CC perception	− 0.062	0.402	− 0.150
Rainfall_var	− 0.415	0.105	− 3.960***
_cons	2.905	1.012	2.870**

Number of obs 385

Prob > chi2 0.000

Pseudo R2 0.2436

*, **, and *** represents level of significant at 10%, 5%, and 1% levels, respectively

**Fig. 6:**
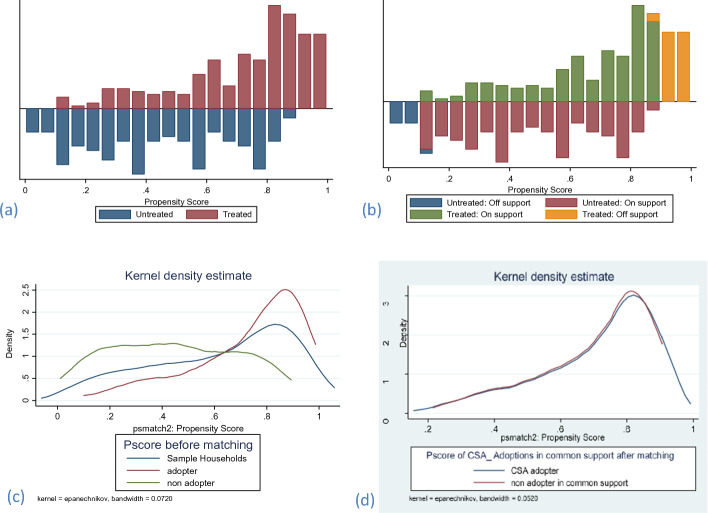
Propensity score distribution and common support region for propensity score estimation: **a** treated and untreated propensity score; **b** propensity score in common support region. Treated on support indicated the individuals in groups who found a suitable match, and treated off support indicated the individuals in the group who did not find a suitable match

The common support condition is executed in the estimation by running matching techniques in the common support region. Figure 6 shows the distribution and common support propensity scores before and after matching. When the following criteria are satisfied, it is deemed that the matching exercise was successful: There should not be any systematic differences in the distribution of the covariates between matching groups; a low pseudo R^2^ is anticipated; a lower standard means bias; and the rejection of significant joint variables after matching (Caliendo & Kopeinig, 2008). The covariate distribution after matching, pseudo *R*^2^ (0.019), and the *p*-value (1.000) of the likelihood ratio show that covariates are balanced across households of adopters and non-adopters.

There is enough overlap between CSA adopters and non-adopters, as shown in Fig. 3’s common support graph. Before matching, there is a roughly 70% disparity between adopters and non-adopters’ propensity score amounts; after matching, the bias was reduced to 4.7%. As explained before, matching considerable variables show significant deference, but after matching, all variables became insignificant and balanced. Additionally, the covariate balancing test results showed that the distribution of covariates among CSA adopters and non-adopters was comparable after matching, as evidenced by the low pseudo R2, insignificant *p*-value, and mean standard bias (< 20%).

### Estimation of the ATE of CSA adoptions

The total effect of CSA on crop income and weekly basis FCS was estimated using alternative matching algorithms such as NNM, kernel matching, and radius matching. The ATT estimation in all three matching algorithms shows that adopting CSA practices positively impacts household crop income and food consumption scores. Table 3 shows the estimated ATEs on the treated (ATT) from CSA adoption matching algorithms. For FCS, the ATT estimation results from NNM, kernel matching, and radius matching are 6.27, 8.15, and 7.47, respectively. Meanwhile, the ATE of an increase in weekly FCS ranges from 6.27 to 8.15 higher for CSA adopters than non-adopters. Tafesse et al. (2020) discovered that the effects of moringa crop adopters on FCS were 6.2 higher than non-adopters in southern Ethiopia. Similarly, CSA adoption has a significant positive impact on household crop income per hectare, as shown in Table 3. The ATEs of CSA adoption on crop income range from 4902.41 to 5116.91 ETB higher than non-adopters. This study’s findings are consistent with Fentie and Beyene (2019), who found that adopting CSA positively impacts household yield and crop income. The findings indicate that farmers could increase crop income and reduce food insecurity by implementing CSA practices and technology. Farmers who use CSA practices consume more diverse food and yield higher income than non-adopters do. Moreover, the positive effects of CSA adoption can improve farmers’ adaptive capacity to climate risks (Bedeke et al., 2019; Tesfay, 2020).

**Table 3:** Average impact of CSA adoption on household income and food security

Outcome variables	Matching algorithms	Treated	Control	ATT	Pseudo-*R*^2^	Standard mean bias	S.E	*z*-value
Income	NNM	15,048.01	9972.94	5057.07	0.027	5.0	1405.34	3.61
	KM	14,875.36	9758.44	5116.91	0.031	4.8	1410.48	3.63
	RM	14,875.36	9972.94	4902.41	0.134	7.1	984.69	4.98
FCS	NNM	43.43	37.15	6.27	0.036	8.0	1.96	3.19
	KM	43.96	35.81	8.15	0.037	4.3	1.06	7.63
	RM	43.72	36.24	7.47	0.134	7.5	0.56	13.18

The scope of this study is the positive and significant average effects of CSA on households’ crop income and FCS, and it considered only the total effects. However, adopting CSA practices and technologies have multiples effects on different farmers’ livelihood systems, environment, market, health, and industry (Kalimba & Culas, 2020; Sardar et al., 2021).

### Sensitivity analysis of treatment effects

The matching approach is the widely used method to estimate ATEs (Karimi et al., 2020). The estimation of the ATE using various matching algorithms is not robust against hidden bias. It is assumed that hidden bias characteristics that go unobserved could impact matching estimation for the treatment and outcome variables (Becker & Caliendo, 2007; Rosenbaum, 2002). Rosenbaum's (2002) proposed abounding approach was used to address such hidden bias. The outcome variable estimated at various critical level value of gamma indicated the *p* critical values are significant, implying that the important covariates which can affect both CSA adoption and outcome variables have been considered in this study. We did not find the critical value of gamma* that questioned the estimated value of ATT (Appendix 1). According to the bounds estimation, the results of ATEs are therefore not sensitive to the presence of the hidden bias. Because this is the only effect of the adoption of CSA practices on household income and food security, our impact estimate of ATT is not sensitive to unobserved selection bias.

## Conclusion and policy implication

The study examined the impact of CSA practices and technological interventions on farm income and food security in rural households in southern Ethiopia. It relied on observational data from household surveys in selected CSVs in southern Ethiopia. The study highlighted CSA measures’ substantial implications on farm income and food security. Smallholder farmers are adopting several CSA innovations to mitigate the negative effects of CC and maintain agricultural productivity. The study’s key findings show that smallholder farmers who implemented CSA measures earned significantly more farm income and improved their food security compared with non-adopters. The contribution of CSA measures on farmers’ income and food security can be strengthened by providing subsidies, extension services, and accurate climate services. This study suggests that promoting and scaling up a portfolio of CSA measures for farmers living in diverse landscapes should be identified and prioritized. In this regard, smallholder farmers’ knowledge and awareness of CC and its impact must be prioritized because it helps to increase the adoption rate of appropriate CSA innovations in their farming system.

The study contributes to the existing literature by providing useful baseline information for future CSA research and policy intervention. However, the study has some limitations. First, the study should rely on cross-sectional farm-level household data due to a lack of baseline data, which limits the investigation of the dynamics of CSA intervention over time. This may affect the estimation results due to the intervention’s spillover effects and unobserved heterogeneity. Second, this study only measured the aggregate effects of selected CSA practices and technologies. However, each CSA practice and technologies have a different level of impact on household welfare, and an independent study is required to provide detailed CSA measures for designing specific interventions. Moreover, future studies need to use panel data collected from both program participants and non-participants before and after implementation of the program intervention to help assess the dynamic nature of CSA interventions and capture possible unobserved heterogeneities.

## Data Availability

The datasets generated during and/or analyzed during the current study are available from the corresponding author on reasonable request.

## Appendix 1

(See Tables

**Table 4:** Rosenbaum bounds for _FCS (*N* = 384 matched pairs)

Gamma	sig +	sig −	t-hat +	t-hat −	CI +	CI −
1	0	0	39.9876	39.9876	39.9876	39.9876
1.25	0	0	39.9876	39.9876	39.9876	39.9876
1.5	0	0	39.9876	39.9876	39.9876	43.7264
1.75	0	0	39.9876	39.9876	39.9876	43.7264
2	0	0	39.9876	43.7264	39.9876	43.7264

*gamma - log odds of differential assignment due to unobserved factors

sig + - upper bound significance level

sig − - lower bound significance level

t-hat + - upper bound Hodges-Lehmann point estimate

t-hat − - lower bound Hodges-Lehmann point estimate

CI + - upper bound confidence interval (a = .95)

CI − - lower bound confidence interval (a = .95)

4 and

**Table 5:** Rosenbaum bounds for log crop income (*N* = 384 matched pairs)

Gamma	sig +	sig −	t-hat +	t-hat −	CI +	CI −
1	0	0	10,500	10,500	9500	11,450
1.25	0	0	9500	11,400	8700	12,500
1.5	0	0	8850	12,250	8000	13,500
1.75	0	0	8325	13,000	7500	14,500
2	0	0	7900	13,750	7100	15,500

*gamma - log odds of differential assignment due to unobserved factors

sig + - upper bound significance level

sig − - lower bound significance level

t-hat + - upper bound Hodges-Lehmann point estimate

t-hat − - lower bound Hodges-Lehmann point estimate

CI + - upper bound confidence interval (a = .95)

CI − - lower bound confidence interval (a = .95)

5).

Income and crop yield changes after CSA adoption.

Changes in income due to CSA adoptions Change in crop yield due to CSA adoption.

## Appendix 2: Selected CSA practices in the study area

Selected CSA practices in the study area.

## Appendix 3: Variable description and measurement used in the model

**Table:**

Variable explanation		Mean	Std. Dev
CSA adoption	1 for adopters, 0 otherwise	0.618	0.486
Climate change perceptions	1 for perceived, 0 otherwise	0.868	0.339
Age	Actual age of the household head in years	50.423	13.461
Education	Level of education in years	3.112	3.854
Family size	Number of family members in the household	7.429	2.451
Family labor	Number of productive labors	3.79	1.08
Gender	1 for male, 0 otherwise	0.805	0.397
Landholding size	Total crop landholding in hectares	0.617	0.466
Farming experiences	Actual farming experience of the household	25.67	13.538
Distance outputmar ~ t	Distance of input and output market in km	4.871	5.7
Access climatetinf ~ n	1 for access to climate information, 0 otherwise	0.771	0.42
Contact extension	Number of annual contacts with extension agents	5.117	12.298
Training received	1 if the farmers had received training, 0 otherwise	0.732	0.443
Access credit	1 if the farmers had access to credit, 0 otherwise	0.286	0.452
Social membership	1 if the farmer was a member of a social group, 0	0.956	0.206
TLU	Tropical livestock unit	2.442	1.868
Income	Estimated annual income in Ethiopian currency	12,571.66	907.48
Soil fertility	1 if a farmer has fertile soil, 0 otherwise	0.63	0.04
Rainfall	Average annual rainfall in mm	1249.1	441.336
Slope of farm plot	1 if farm plot is steep slope, 0 otherwise	0.56	0.032

## Appendix 4: Results of the Heckman probit selection model

**Table:**

Variables	Outcome model				Selection model			
	Regression		Marginal value		Regression		Marginal value	
	Coefficient	*z*-value	Coefficient	*z*-value	Coefficient	*z*-value	Coefficient	*z*-value
Age	0.012	1.93	0.012	1.93	0.026	2.87	0.003	2.45
education	0.044	4.98	0.214	2.35	0.154	16.61	0.026	5.53
Family size	0.031	1.83	0.031	4.39				
Gender	0.103	0.89	0.103	0.89	0.016	0.08	0.003	0.48
landholding_size	0.160	8.46	0.161	2.17				
farming_experiences	0.024	4.19	0.039	6.90	0.028	4.19	0.036	2.85
access_extension	0.137	3.43	0.137	4.58				
distance_outputmarket	− 0.012	− 1.71	− 0.012	− 1.71				
access_climatetinformation	0.402	5.99	0.202	6.79	1.084	5.99	0.231	8.56
contact_extension	0.213	6.20	0.013	2.93				
Training_received	0.224	2.21	0.224	2.21				
access_credit	0.001	0.01	0.001	0.01				
Social_member	0.206	20.29	0.106	2.59	0.620	1.70	0.005	2.72
TLU	0.013	5.78	0.013	5.77				
Annual income	0.235	2.68	0.235	2.68				
Soil fertility	0.135	4.71	0.135	1.37				
slop_farmplot	− 0.012	− 0.14	− 0.012	− 0.14				
rainfall_var					0.069	1.97	0.020	2.21
Constant	0.490	2.65			0.121	2.79		
Total observation 385								
Censored 79								
Uncensored 306								
Wald chi-square (zero slopes) 82.45, *p* < 0.001								
Wald chi-square (independent equation)	10.25, *p* < 0.001							

Source: Authors’ computation based on survey data 2021.
